# Differences in oxygen uptake but equivalent energy expenditure between a brief bout of cycling and running

**DOI:** 10.1186/1743-7075-3-1

**Published:** 2006-01-03

**Authors:** Christopher B Scott, Nathanael D Littlefield, Jeffrey D Chason, Michael P Bunker, Elizabeth M Asselin

**Affiliations:** 1 University of Southern Maine, Department of Sports Medicine, 37 College Ave. Gorham, ME 04038, USA

## Abstract

**Background:**

We examined aerobic and anaerobic exercise energy expenditure and excess post-exercise oxygen consumption (EPOC) between a 250 Watt, 1-minute bout of cycling and uphill treadmill running.

**Methods:**

Fourteen active to well-trained subjects volunteered for the investigation (VO_2 _max: 57.0 ± 12.9 ml·kg·min^-1 ^cycle; 59.3 ± 13.7 ml·kg·min^-1 ^run; p = 0.44). Anaerobic energy expenditure was estimated from △blood lactate. Statistical analysis was completed using a paired t-test (mean ± SD).

**Results:**

Perceived exertion did not differ between exercise bouts (14.0 ± 2.3 cycle; 13.2 ± 2.1 run; p = 0.29). Exercise oxygen uptake was significantly greater for running (41.4 ± 6.9 kJ) compared to cycling (31.7 ± 7.7 kJ) (p = 0.0001). EPOC was not different between cycling and running (p = 0.21) so that exercise oxygen uptake + EPOC was greater for running (103.0 ± 13.5 kJ) as compared to cycling (85.4 ± 20.2 kJ; p = 0.008). Anaerobic energy expenditure was significantly greater for cycling (32.7 ± 8.9 kJ) versus running (22.5 ± 11.1 kJ) (p = 0.009). Aerobic + anaerobic exercise energy expenditure (cycle 64.3 ± 12.2 kJ; run 63.9 ± 10.1 kJ) (p = 0.90) and total energy expenditure (including EPOC; cycle 118.0 ± 21.8 kJ; run 125.4 ± 19.1 kJ; p = 0.36) were similar for cycling and running.

**Conclusion:**

Oxygen-only measures reveal discrepancy in energy expenditure between cycling and uphill running. Measurements of exercise oxygen uptake, △blood lactate and a modified EPOC promote the hypothesis of a similarity in exercise and total energy expenditure between 1-minute work-equivalent bouts of cycling and uphill running.

## Background

The conversion of oxygen uptake into an estimate of metabolic heat production is the most widely used method of determining the energy expenditure of rest and steady-rate exercise. Yet the presence of an oxygen deficit during brief, intense, non-steady state work suggests that this type of activity can not be accurately quantified with a measurement of exercise oxygen uptake. Likewise, the excess post-exercise oxygen consumption (EPOC) does not provide a valid representation of rapid glycolytic ATP re-synthesis during intense exercise [1,2]. Thus, oxygen-only measurements have the potential to underestimate energy expenditure for brief, heavy to severe exercise that contains a significant anaerobic energy expenditure component.

Cycling and running are described as aerobic activities because they both utilize the large muscle groups of the lower body in repeated and rhythmic contractions. It is apparent however that differences in muscle recruitment patterns between these two activities can invoke different aerobic and anaerobic contributions to exercise energy expenditure. For example, oxygen uptake measurements taken over 15–30 minutes of exercise revealed that treadmill running had significantly greater oxygen uptake and, presumably, greater energy expenditure as compared to cycling [3-5]. Even so it has also has been shown that the post-exercise energy expenditure after 30 minutes of running and cycling were not different suggesting that the metabolic demands of these discrepant exercises may have been similar [5]. In fact, submaximal and maximal cycling produces blood lactate concentrations that are larger as compared to treadmill running and this may signify a greater anaerobic energy expenditure component to cycling as compared to running [6]. Based on these findings we asked the question, would equivalent bouts of cycling and running have similar energy expenditure if the aerobic and anaerobic energy expenditure were considered independent components of the measurement of total energy expenditure [7]?

A standardized means of comparison among diverse exercise modes is an area of concern. Previous studies that have examined energy expenditure among different exercise modes have attempted comparisons that promote "similarity" in work intensity. For example, exercise intensity has been based on self-selection [3,4], perceived exertion [8], percentage of maximum heart rate [9] or a predetermined percentage of VO_2 _max [5,8]. In the current investigation an attempt was made to equate work output (250 Watts in 1 minute) between non-steady state cycling and uphill running. We hypothesized that estimates of both aerobic and anaerobic ATP turnover, as opposed to oxygen-only measurements, would indicate no difference in exercise and total energy expenditure between work-equivalent 1-minute bouts of intense cycling and uphill running.

## Methods

### Subjects

The study was approved by the University of Southern Maine's Institutional Review Board. Informed consent was obtained from fourteen physically active to well trained volunteers (179.6 ± 8.0 cm; 77.5 ± 14.2 kg; 28.6 ± 9.4 years; 13 male, 1 female).

### Data collection

Subjects randomly completed two maximal exercise tests to exhaustion on separate days. The treadmill max test was completed to volitional exhaustion using a standard Bruce protocol (Trackmaster treadmills, Carrollton, TX). The maximal cycling test was completed to volitional exhaustion with a Diamondback road bicycle mounted on a Velodyne ergometer (Velodyne Sports, Laguna Hills, CA). The cycle max test was started at 50 Watts with an additional 50 Watts added every 2-minutes until 250 Watts was reached; at this point the increase was 25 Watts every 2-minutes. Gas exchange was collected in 15-second sampling periods throughout the maximal and 1-minute exercise tests with a Parvomedics metabolic cart (Sandy, UT). Blood lactate concentrations were collected in duplicate from a finger-stick (droplet of whole blood) at rest and 2-minutes into a seated recovery after the exercise tests (Lactate Pro, Arkray, Inc., Kyoto, Japan). A pilot project determined peak lactate levels for these activities occurred at 2-minutes post-exercise (see also [10]).

### Exercise protocols

Subjects reported to the laboratory twice on separate days, at least 3-hours post-prandial, and were randomly assigned to either the brief cycle or run protocol. Before exercise testing a 5-minute seated (on the bike) or standing (on the treadmill) resting oxygen uptake measurement was recorded. Resting energy expenditure was based on the respiratory exchange ratio (RER) and was subtracted from all exercise oxygen uptake and EPOC measures.

The power output for cycling is measured in Watts. We estimated power output for treadmill running based on a standard conversion of the vertical work, body weight and running speed. Both tests were designed to elicit a power output of 250 Watts (i.e., work rate) over the course of 1-minute (i.e., work output). This absolute workload was selected to ensure all subjects were working intensely, that they could complete the 1-minute of exercise and to attempt to equate work output and time between exercise modes (selecting a work rate as a relative percentage of VO_2 _max has the potential to influence individual exercise time, further influencing EPOC and energy expenditure). For cycle exercise the Velodyne was pre-set at 250 Watts. Subjects began pedaling on command at 60 rpm's. Treadmill exercise was conducted at a 10% grade with a speed that elicited 250 Watts based on subject weight as:

meters·min^-1 ^= [(1530 kg-m·min^-1^) (body weight in kg)^-1 ^(0.1) ^-1^]

(e.g., a subject weighing 85 kg would run at 180 meters·min^-1^; 250 Watts = 1530 kg-m·min^-1^). Exercise energy expenditure for aerobic and anaerobic metabolism was converted as 1 liter of O_2 _= 21.1 kJ. Upon completion of the 1-min work period, subjects were immediately seated in a chair next to the cycle or treadmill and EPOC was recorded until it fell below the respective 5-min resting O_2 _uptake measurement. EPOC was calculated as 1 liter of O_2 _= 19.6 kJ to exclude rapid glycolytic ATP re-synthesis as part of the conversion of oxygen uptake into energy expenditure; in this regard EPOC represented aerobic energy expenditure only [2,7]. Anaerobic glycolytic energy expenditure (i.e., rapid glycolytic ATP re-synthesis) was estimated using blood lactate (use of the "anaerobic" ATP/PC stores was assumed to be accounted for as part of EPOC [7]). Blood lactate measures (mmoles) were converted to oxygen equivalent values as 3 ml O_2_·kg^-1 ^body weight per mmol of △blood lactate [11]. △blood lactate was obtained by subtracting resting from peak lactate concentrations [11].

### Data analysis

Aerobic exercise energy expenditure, anaerobic energy expenditure and acute recovery energy expenditure were compared between cycling and uphill treadmill running with a standard 2-tailed paired t-test.

## Results

All measurements are reported as mean ± SD. VO_2 _max did not differ between cycle and treadmill testing (57.0 ± 12.9 ml·kg·min^-1 ^cycle; 59.3 ± 13.7 ml·kg·min^-1 ^run; p = 0.44). Peak watts during the max test also did not differ (measured 279 ± 33 Watts cycle; estimated 281 ± 65 Watts treadmill). Subject ratings of perceived exertion (RPE) were not different between exercise tests (14.0 ± 2.3 cycle; 13.2 ± 2.1 run; p = 0.29) (13 = "somewhat hard"; 15 = "hard"). Absolute energy expenditure values are shown in Table 1. Aerobic plus anaerobic exercise energy expenditure was similar for cycling (64.3 ± 12.2 kJ) and running (63.9 ± 10.1 kJ) (p = 0.90). Aerobic exercise energy expenditure was significantly lower for cycling as compared to running (31.7 kJ vs 41.4 kJ, respectively; p = 0.0001). Anaerobic exercise energy expenditure was significantly larger for cycling as compared to the run (32.7 kJ vs 22.5 kJ, respectively; p = 0.009). EPOC values were not statistically different (53.7 kJ cycle vs 61.5 kJ run; p = 0.21). Aerobic exercise energy expenditure and EPOC values were significantly less for cycling as compared to running (85.4 kJ vs 103.0 kJ, respectively; p = 0.008). Total energy expenditure that included aerobic and anaerobic exercise energy expenditure along with EPOC was not statistically different (118.0 kJ cycle vs 125.4 kJ run; p = 0.36).

**Table 1:** Absolute energy expenditure between cycling and uphill running.

Component	Cycling	Running	Sig. (p)
VO_2 _max (ml·kg·min^-1^)	57.0 ± 12.9	59.3 ± 13.7	0.44
Exercise O_2 _(kJ)	31.7 ± 7.7	41.4 ± 6.9	0.0001
△blood lactate (kJ)	32.7 ± 8.9	22.5 ± 11.1	0.009
Exercise O_2 _+ △blood lactate (kJ)	64.3 ± 12.2	63.9 ± 10.1	0.90
EPOC (kJ)	53.7 ± 21.6	61.5 ± 11.7	0.21
Exercise O_2 _+ EPOC (kJ)	85.4 ± 20.2	103.0 ± 13.5	0.008
Total Energy Expenditure (kJ)	118.0 ± 21.9	125.4 ± 19.2	0.36

Mean ± SD. See methods section for energy expenditure conversions. Total energy expenditure equals exercise oxygen uptake + △blood lactate + modified EPOC. Power output was preset at 250 Watts for each 1-minute test. Peak power outputs during max testing were 279 ± 33 Watts for cycling and an estimated 281 ± 65 Watts for uphill treadmill running (highest treadmill work rate achieved). Resting oxygen uptake was subtracted from exercise oxygen uptake and EPOC measurements. Resting energy expenditure was not different between trials (9.3 ± 1.6 kJ min^-1^, cycle; 9.5 ± 2.1 kJ min^-1^, run) (p = 0.62). Respiratory exchange ratio (RER) at rest was not different between trials (0.84 ± 0.05, cycle; 0.83 ± 0.06, run) (p = 0.52).

Relative energy expenditure values are shown in Table 2. Percent aerobic contributions (exercise O_2 _uptake + EPOC) were significantly lower for cycling compared to running (72.1% vs 82.8%, respectively; p = 0.0001). Percent aerobic exercise energy expenditure was significantly less for cycling as compared to running (27.7% vs 34.0%, respectively; p = 0.02). Percent anaerobic energy expenditure was significantly greater for cycling as compared to running (27.9% vs 17.2%, respectively; p = 0.0001). Percent energy expenditure contributions for EPOC were not statistically different between tests (44.4% cycle vs 48.8% run; p = 0.08).

**Table 2:** Relative energy expenditure between cycling and uphill running.

Component	Cycling	Running	Sig. (p)
Exercise O_2_	27.7% ± 7.4	34.0% ± 8.0	0.02
△blood lactate	27.9% ± 6.9	17.2% ± 7.9	0.0001
EPOC	44.4% ± 10.4	48.9% ± 4.0	0.08
Exercise O_2 _+ EPOC	72.1% ± 6.9	82.8% ± 7.9	0.0001

Mean ± SD

## Discussion

As with previous investigations using longer duration exercise and various standards of comparison [3-6], our data reveal that exercise oxygen uptake is lower and blood lactate higher for a brief bout of intense non-steady state cycling as compared to uphill running. Previous research also has shown that excess post-exercise oxygen consumption (EPOC) is not different between comparable bouts of longer duration cycling and uphill running even though running had greater aerobic exercise energy expenditure [5]; this also was true for the current study. In agreement with our hypothesis, the data suggest that when a measure of aerobic energy expenditure and an estimate of anaerobic energy expenditure are both used to independently interpret energy transfer, then exercise and total energy expenditure between work-equivalent 1-minute bouts of cycling and uphill running are similar. Additional studies are needed to determine if this finding extends to lower intensity, longer duration exercise.

Heavy to severe exercise promotes rapid glycolytic ATP re-synthesis [12-17]. Moreover, measurements of both lactate production [18] and lactate oxidation [2] using simultaneous direct and indirect calorimetry have conclusively demonstrated that rapid anaerobic glycolytic ATP re-synthesis can not be represented by a measurement of oxygen uptake during or after exercise. Even so, investigations that estimate rapid glycolytic ATP re-synthesis rarely add this component to a measure of EPOC to obtain a measurement of total energy expenditure; this may be because of the reluctance of researchers to dismiss the traditional practice of having rapid glycolytic ATP re-synthesis quantified as part of an oxygen uptake measurement, as the oxygen debt hypothesis proposed [2,7]. By keeping this aspect of anaerobic metabolism separate from aerobic measurements we found that total energy expenditure contained a rapid glycolytic ATP re-synthesis component that was 28% for cycling and 17% for running (Table 2).

If oxygen uptake were the only means of measuring energy expenditure for the brief, intense and equivalent work in the current study then it would be falsely concluded that cycling was the more efficient and running the more "expensive" exercise (Table 1). However, cycling appears to recruit a different muscle mass as compared to running that can result in an accelerated glycogenolysis with lactate production that plays a significant role in total ATP re-synthesis [6]. Our data suggest that the difference in motor unit recruitment patterns between equivalent bouts of cycling and uphill running are more likely to influence the extent of aerobic and anaerobic energy transfer rather than exercise and total energy expenditure. We propose that a measurement of exercise oxygen uptake (i.e., aerobic energy expenditure) along with a reasonable estimate of anaerobic glycolytic energy expenditure (i.e., △blood lactate) may provide a better comparison of energy expenditure between unlike exercise modes.

This study is certainly not without limitations as assumptions were made about equivalent work output, metabolic and work efficiency, and the inherent problems of converting blood lactate into an estimate of anaerobic energy expenditure. Blood lactate is a questionable marker in the quantification of rapid glycolytic ATP re-synthesis because it is a dynamic metabolite that is produced and removed at different rates. It also has been well demonstrated that muscle and/or blood lactate concentrations can be highly variable both within and among subjects [12-17,19]. However, as mentioned throughout this manuscript, the use of oxygen-only measurements also introduces considerable error into the quantification of energy expenditure for brief, intense, non-steady state exercise. Indeed, we have shown that in exercise fueled by a large proportion of rapid glycolytic ATP re-synthesis, the largest potential for error in quantifying and interpreting total energy expenditure for brief intense activity occurred not with the estimate of anaerobic energy expenditure, but when the anaerobic energy expenditure component is excluded or omitted from the estimate of total energy expenditure [19]. As an indirect measure, blood lactate levels after sprinting have been shown to provide a reasonable estimate of rapid muscle glycolytic ATP turnover [12]. In fact, a favorable case has been made for the use of blood lactate as a reasonable estimate of anaerobic energy expenditure that has persisted for well over 30 years [11,16]. Our methodology included anaerobic ATP re-synthesis as part of glycogen metabolism during the exercise oxygen uptake measurement (1 lO_2 _= 21.1 kJ) but we dismissed this anaerobic component from the EPOC measurement (1 lO_2 _= 19.6 kJ) to avoid oxygen debt-type interpretations of rapid glycolytic ATP turnover [2,7]. Rapid glycolytic ATP re-synthesis during exercise (that exceeds exercise oxygen uptake) was estimated independently as 3 ml O_2_·kg^-1 ^per mmol of △blood lactate (i.e., lactate was converted into oxygen equivalent units then as 1 lO_2 _= 21.1 kJ) [11]. This blood lactate conversion was derived from running [16] but has been reported to be similar for several forms of exercise [11]. A conversion for cycling has been reported at 5.2 ml O_2_·kg^-1 ^per mmol of blood lactate but this was not based on △blood lactate [14] and thus may lack credibility [11].

In terms of work efficiency it has been shown that cycling and treadmill walking can be similar but caution should be applied as comparisons are heavily influenced by speed and incline [20]. Treadmill running also contains a horizontal along with a vertical power output component that may increase the total work output as compared to cycling; we did not account for this horizontal work component. While potential differences in the horizontal and vertical components of power output along with metabolic and work efficiency are certainly possible between an intense bout of cycling and uphill running, it did not promote significant discrepancy in perceived exertion or recovery energy expenditure (EPOC) between the work bouts so that subjects also appeared to have worked at the same relative exercise intensity. We therefore argue that work output along with the metabolic and work efficiencies were not significantly different between cycling and uphill treadmill running in the current investigation, though exercise oxygen uptake indicated the opposite (Table 1).

## Conclusion

Oxygen-only measures reveal discrepancy in energy expenditure between cycling and uphill running. Measurements of exercise O_2 _uptake, △blood lactate and a modified EPOC promote the hypothesis of a similarity in exercise and total energy expenditure between 1-minute work-equivalent bouts of cycling and uphill running.

## Authors' contributions

CBS conceived and designed the study and wrote the final manuscript. NDL was involved in study design, anaerobic and aerobic data collection and quantification during the submax tests and helped draft the final manuscript. JDC was responsible for study design along with aerobic and anaerobic data collection during submax testing. MPB was primarily responsible for data collection during max testing and also assisted with submax test data collection. EAM performed the pilot work on blood lactate collection, study design and also assisted with data collection.
